# The Association Between Midlife Education and Depressive Symptoms in Late Life: The AGES-Reykjavik Study

**DOI:** 10.1093/geroni/igab046.1098

**Published:** 2021-12-17

**Authors:** Milan Chang, Chiharu Nishizuka, Hrafnhildur Eymundsdottir, Sigurveig Sigurdardottir, Alfons Ramel, Vilmundur Gudnasson, Palmi Jonsson

**Affiliations:** 1 The Icelandic Gerontological Research Institute, Reykjavik, Hofuoborgarsvaoio, Iceland; 2 Faculty of Social Science, Reykjavik, Hofuoborgarsvaoio, Iceland; 3 The Icelandic Gerontological Research Institute, Gardabaer, Hofuoborgarsvaoio, Iceland; 4 University of Iceland, Reykjavik, Hofuoborgarsvaoio, Iceland; 5 University hospital Landspitali, Reykjavik, Hofuoborgarsvaoio, Iceland

## Abstract

BACKGROUND: Disability and depression are associated with cumulative health adversities such as socioeconomic status (SES), nutrition, medical care, and education among older adults. However, there is little evidence on the longitudinal association between mid-life education level with a disability and depressive symptoms in older adults in Iceland. The aim of the study was to examine the association between mid-life education and prevalence of activity of daily living (ADL) dependency and high depressive symptoms in late-life. METHODS: A large community-based population residing in Reykjavik, Iceland (n=4991, 57.3% women, 76.9±5.8 yrs) participated in a longitudinal study with an average of 25 years of follow-up. Mid-life education was categorized into 4 groups (primary, secondary, college, and university). ADL dependency and high depressive symptoms were assessed on average 25 (±4) years later. The 5-item ADL dependency score ranged between 0 (no difficulty) and 18. Depressive symptoms were assessed by the 15-item Geriatric Depression Scale (GDS). RESULTS: After controlling for demographic and health-related risk factors, those with higher education at mid-life were significantly less likely to have high depressive symptomatology (6 or higher GDS scores, Odds Ratio (OR) = 0.65, 95% Confidence Interval (CI): 0.52 ~ 0.82, P < 0.0001). However, mid-life education was not associated with ADL dependency in later life. CONCLUSION: Our study shows that mid-life education is associated with depressive symptoms 25 years later, while no association found with ADL dependency among Icelandic older adults.

